# Factors related to children’s caries: a structural equation modeling approach

**DOI:** 10.1186/1471-2458-14-1071

**Published:** 2014-10-15

**Authors:** Rong Min Qiu, Edward CM Lo, Qing Hui Zhi, Yan Zhou, Ye Tao, Huan Cai Lin

**Affiliations:** Department of Preventive Dentistry, Guanghua School of Stomatology, Guangdong Provincial Key Laboratory of Stomatology, Sun Yat-sen University, 56 Ling Yuan Road West, Guangzhou, Guangdong Province 510055 China; Department of Pediatric dentistry, College of Stomatology, Guangxi Medical University, Nanning, China; Dental Public Health, Faculty of Dentistry, University of Hong Kong, Hong Kong, China

**Keywords:** Child, Caregiver, Caries, Oral health knowledge, Attitude, Practice

## Abstract

**Background:**

Dental caries among preschool children is highly prevalent in many less-developed countries.

**Methods:**

A model which explored the factors related to children’s dental caries was tested in this study using structural equation modeling. Caregivers of children aged 5 years were surveyed on their socioeconomic status, and their oral health knowledge, attitudes and practices. In addition, information on their children’s oral health practices, dental insurance and dental service utilization were collected. Examination of caries was conducted on all children who returned fully completed questionnaires.

**Results:**

The results showed that socioeconomic factors influenced children’s oral health practices through the impact of caregivers’ oral health knowledge and practices; that caregivers’ oral health knowledge affected children’s oral health practices through the influence of caregivers’ oral health attitudes and practices; and finally, that children’s oral health practices were linked directly to their caries.

**Conclusion:**

The findings have important applications for promoting policies aimed at advancing children’s oral health.

## Background

Despite great improvement in the oral health of various populations world-wide over the last two decades, caries among preschool children still remain highly prevalent in many communities, particularly among underprivileged groups in developed and developing countries [1]. Data from the Third National Oral Health Survey in China showed that the prevalence of caries among preschool children aged 5 years was as high as 66% and that the mean number of decayed, missing and filled teeth was 3.5 [2]. For the development of effective prevention strategies, it is important to find out the risk factors for children’s dental caries.

Dental caries is linked to preventable and lifestyle-related risk factors and its control should focus on reducing disease risk. Additionally, it is suggested that in the risk-factor approach to promoting oral health, more attention should be paid to the distal sociocultural and environmental factors as well as the roles of intermediate and modifiable risk behaviors [1]. It is also alleged in the Liverpool declaration of promoting oral health in the 21st century that countries should provide evidence-based programs that promote healthy lifestyles and reduce the risk factors common to oral and general chronic diseases [3]. Based on the results of previous studies and from the standpoint of public health, in this study, we studied the socioeconomic factors and oral health knowledge, attitudes, and behaviors related to children’s dental caries.

Although many factors related to dental caries have been identified in previous studies using multiple regression models and associations have been shown [4, 5], it is not clear whether these factors lead to caries directly or indirectly. Therefore, the complex processes for caries should be explored to distinguish between correlational and causal associations, and targeting at these associations can form the basis for effective interventions to improve population oral health and reduce oral health inequalities [6]. A complex model that simultaneously evaluated the multidimensional behavioral pathways that led to ethnic and socioeconomic disparities in oral health was tested in Singaporean preschool children aged 3–6 years through structural equation modeling (SEM) [7]. However, such a complex model has not been tested in Chinese preschool children.

**Figure 1:**
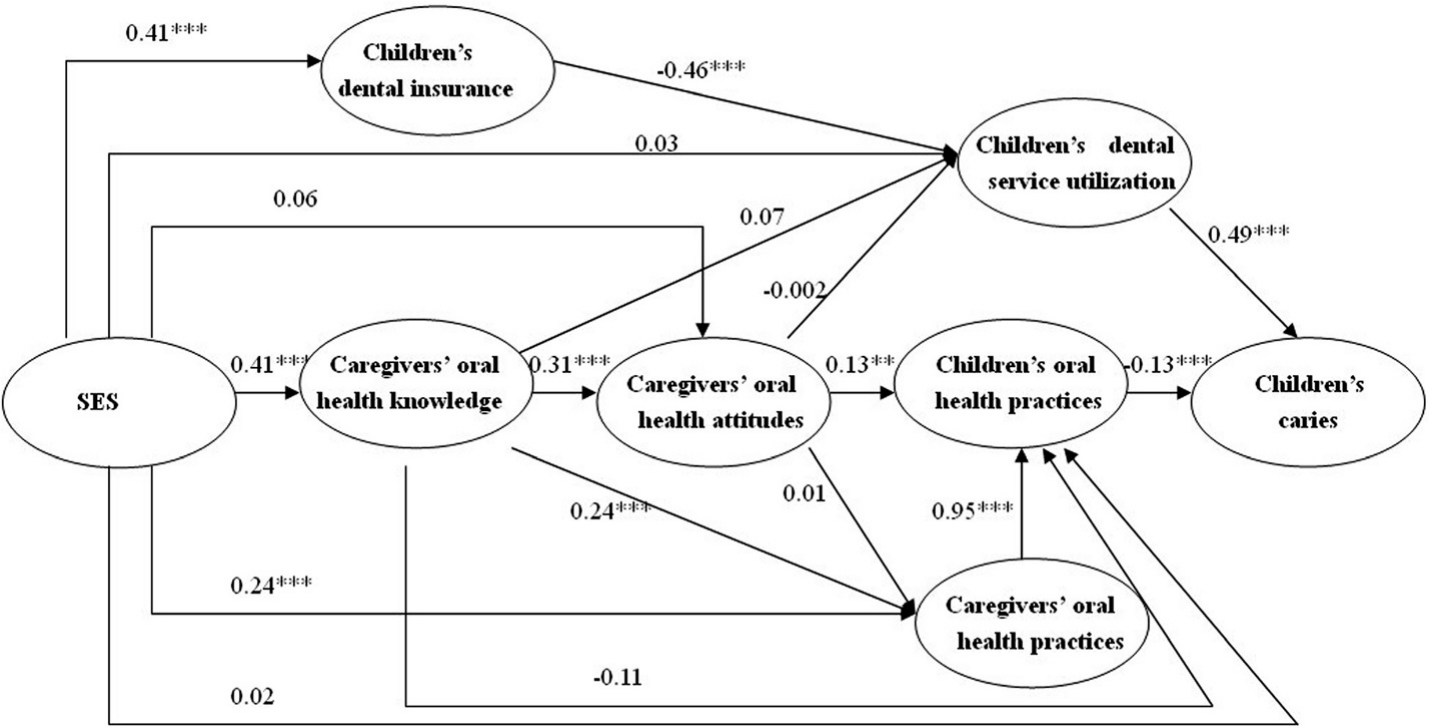
Path diagram of the primary model (The level of significance for the path coefficient was set at 0.05; * ***p*** <0.05; ** ***p*** <0.01; *** ***p*** **<0.001).**

In this study, to explore the factors related to children’s dental caries and how these factors influence the disease, a multi-structural model (Figure 1) was hypothesized based on the theoretical framework about the relationships among dental health knowledge, attitude, practice and dental caries [7], and this model was tested using SEM. It was hypothesized that socioeconomic status affected caregivers’ oral health knowledge, which in turn influenced their own attitudes, then impacted on their own and their children’s oral health practices, and finally impacted on children’s dental caries. Simultaneously, socioeconomic status also affected children’s dental insurance coverage and indirectly their dental service utilization. Additionally, the direct impacts of socioeconomic conditions on caregivers’ attitudes, their own and their children’s oral health practices, and children’s dental attendance were also evaluated with regard to children’s dental caries.

## Methods

### Study subjects

A cross-sectional study was conducted from August to December 2011 in 24 randomly selected public and private kindergartens in Guangzhou, China, with 1440 preschool children aged 5 years and their caregivers. A caregiver could be the child’s parent or grandparent, who was mainly in charge of the child’s daily lives. Ultimately, 1332 children participated in this study, giving a response rate of 92.5%. Details about sampling and subject recruitment were described previously [8].

Before the study, ethical approval from the Ethics Committee of the Guanghua School of Stomatology, Sun Yat-sen University, and written informed consent for participation in the study from the children’s caregivers were obtained.

### Questionnaire

A questionnaire study was conducted on the children’s caregivers. Factors included in the questionnaire were: socioeconomic status (SES), caregivers’ oral health knowledge, attitudes and practices, children’s oral health practices, dental insurance and dental service utilization.

SES was measured by mother’s education and occupation, father’s education and occupation, and family income. Caregivers’ oral health practices included frequency of sugary snack intake and tooth brushing frequency. The children’s oral health practices included frequency of sugary snack intake, tooth brushing frequency and the age at which the child began to brush his/her teeth. The children’s dental insurance was explored with one question on whether the child had dental insurance, and the children’s dental utilization was measured by one question on whether the child had visited a dentist. SES, caregivers’ oral health practices, children’s oral practices, dental insurance and dental service utilization were treated as categorical variables.

Caregivers’ oral health knowledge was measured with four questions about the causes and prevention of tooth decay and periodontal disease, which had been used in a previous study [9]. For example, for the causes of tooth decay, a question was set, “Which of the following answer(s) do you think as the causes of dental caries”. A score of 1 was given for each correct answer to the question, and incorrect or “don’t know" answers were scored 0. A maximum of four correct answers were accepted for each question, and so the caregiver could score up to 4 points per question. The overall oral health knowledge score was the sum of the scores of the four questions, which could range from 0 to 16, with higher scores indicating better oral health knowledge. Caregivers’ oral health attitudes were measured with eight questions on dental health beliefs and on the importance of oral health, retaining natural teeth, and the use of dental service, which had been also used in a previous study [9]. The response to each statement was “agree”, “disagree”, or “neither”. A dental attitude score was constructed by counting the total number of statements to which the caregiver showed a positive attitude. The final score could range from 0 to 8, with higher scores indicating a more positive attitude toward oral health. Caregivers’ oral health knowledge and attitude scores were treated as continuous variables.

### Clinical Examination

Examination of the children’s dental caries status was conducted by a trained examiner. Record of the number of decayed, missing and filled primary teeth (dmft) was based on the criteria recommended by the World Health Organization [10]. The children’s caries status was measured by as continuous variable.

### Data Analysis

Data analysis was first carried out using the SPPS for windows version 16.0 software to analyze the variables. Validity of the hypothesized model was tested with confirmatory structural equation modeling using the statistical software Lisrel 8.8.

First, measurement models were tested to determine the factor structure for the latent variables, such as SES, caregivers’ oral health knowledge, attitudes and practices, children’s oral health practices, dental insurance coverage and dental service utilization. The observed variables were defined as the formative items of their constructs in the measurement model (Tables 1,2). Second, structural equation model was applied to test the validity of the hypothesized model. Since some observed variables were categorical, and other observed variables including the children’s caries were continuous, generally weighted least squares was used as the estimation method.

**Table 1:** **Frequency distribution of the observed categorical variables**

Latent variables	Observed variables	n	%
SES	Mother’s education		
	≤ high school graduated	727	54.6
	≥ college graduated	605	45.4
	Mother’s occupation		
	Unemployed	248	18.6
	Employee/non-professional	794	59.6
	Employer/professional	290	21.8
	Father’s education		
	≤ high school graduated	677	50.8
	≥ college graduated	655	49.2
	Father’s occupation		
	Unemployed	52	3.9
	Employee/non-professional	846	63.5
	Employer/professional	434	32.6
	Family monthly income (per-capital)		
	< 2000 RMB	262	19.7
	2000-4999 RMB	546	41
	≥5000 RMB	524	39.3
Caregivers’ oral health practices	Caregivers’ frequencies of sugary snack intake		
	≥ once/day	499	37.5
	< once/day	833	62.5
	Caregivers’ frequencies of toothbrushing		
	≤ once/day	405	30.4
	≥ twice/day	927	69.6
Children’s oral health practices	Children’s frequencies of sugary snack intake		
	≥ once/day	661	49.6
	< once/day	671	50.4
	Children’s frequencies of toothbrushing		
	≤ once/day	896	67.3
	≥ twice/day	436	32.7
	Age of beginning to brush teeth		
	≥ 4 yrs old	394	29.6
	3 yrs old	473	35.5
	≤ 2 yrs old	465	34.9
Children’s dental insurance	Children’s dental insurance		
	No	1056	79.3
	Yes	276	20.7
Children’s dental service utilization	Children’s dental service utilization		
	No	923	69.3
	Yes	409	30.7

**Table 2:** **Scores of the observed continuous variables**

Latent variables	Observed variables	Mean	SD
Caregivers’ oral health knowledge	Score for cause of caries	2.5	1.1
	Score for prevention of caries	2.7	1.1
	Score for cause of periodontitis	1.9	1.2
	Score for prevention of periodontitis	2.0	1.2
Caregivers’ oral health attitudes	Caregivers’ oral health attitudes	6.2	1.4
Children’s caries	Children’s dmft	3.8	4.5

The magnitude and significance of the hypothesized links were evaluated with the *β* and *p*-values, and a *p*-value less than 0.05 was considered statistically significant. The comparative fit index (CFI), the normed fit index (NFI), the root mean square error of approximation (RMSEA), the goodness of fit index (GFI), and the adjusted goodness of fit index (AGFI) were used to measure the goodness of fit of the model. CFI and NFI values ≥0.90, an RMSEA ≤0.06, and GFI and AGFI values ≥0.95 served as criteria for the model’s fitness [11, 12].

## Results

The frequency distributions and the scores for the observed variables are displayed in Tables 1 and 2, respectively.

The hypothesized relationships between the independent factors and children’s dental caries status were represented in the exploratory path model that was tested in this study, and the pathway parameters are displayed in Figure 1. Here, it was shown that children’s dental visit history was positively associated with children’s caries status, which indicated that children with prior dental visit were likely to have more dental caries. In our previous study in Guangdong, China, children were more likely to utilize dental services mainly for dental problems rather than for regular check-ups, and 85% of the children visited their dentists only for fixing dental problems [8]. Therefore, in this study, children’s dental service utilization was probably not influencing the children’s dental caries and was discarded from the model. Considering that dental insurance coverage only indirectly influences children’s oral health through dental service utilization [13], it is logical to remove children’s dental insurance from the model, too.

**Figure 2:**
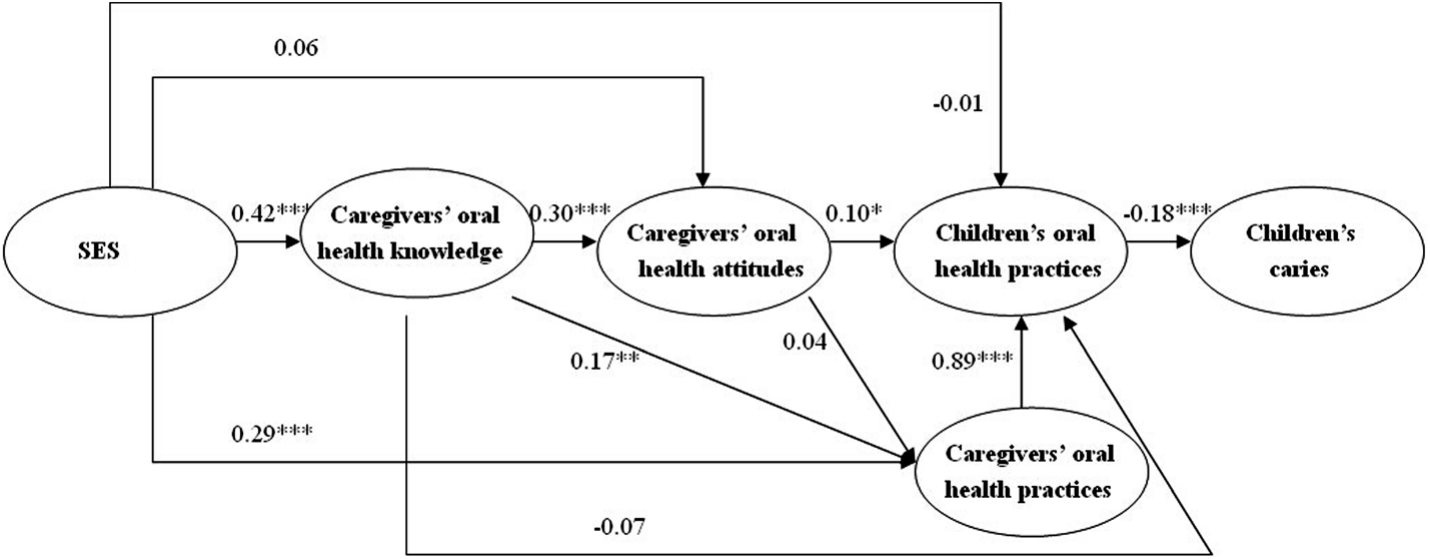
Path diagram of the revised model (The level of significance for the path coefficient was set at 0.05;* ***p*** <0.05; ** ***p*** <0.01; *** ***p*** **<0.001).**

After children’s dental service utilization and dental insurance coverage were removed from the model, a revised model was tested with the data, and the hypothesized relationships are shown in Figure 2. In this model, children’s oral health practices were negatively associated with dental caries (*β* = −0.18, *p* < 0.001), meaning that children who had better oral health practices were likely to have fewer carious teeth. Additionally, children’s oral health practices were positively linked to their caregivers’ oral health attitudes and practices (*β* = 0.10, *p* < 0.05 and *β* = 0.89, *p* < 0.001, respectively) but not directly influenced by SES or by caregivers’ oral health knowledge (*β* = −0.01, *p* > 0.05 and *β* = −0.07, *p* > 0.05, respectively). SES had direct effects on caregivers’ oral health knowledge and their oral health practices (*β* = 0.42, *p* < 0.001 and *β* = 0.29, *p* < 0.001, respectively), but no significant association was found between SES and caregivers’ oral health attitudes (*β* = 0.06, *p* > 0.05). Furthermore, caregivers’ oral health knowledge was positively and directly linked to their oral health attitudes and practices (*β* = 0.30, *p* < 0.001 and *β* = 0.17, *p* < 0.01, respectively). The fit indices indicated good data representation for the full sample (NFI = 0.90, CFI = 0.91, RSEMA = 0.064, GFI = 0.98, AGFI = 0.97). Loading of the observed variables for their latent variables in the final model was showed in Table 3.

**Table 3:** **Loading of the observed variables for their latent variables in the final model**

dmft	Observed variables	Factor loading
SES	Mother’s education	0.95
	Mother’s occupation	0.45
	Father’s education	0.84
	Father’s occupation	0.41
	Family monthly income (per-capital)	0.37
Caregivers’ oral health knowledge	Score for cause of caries	0.63
	Score for prevention of caries	0.76
	Score for cause of periodontitis	0.76
	Score for prevention of periodontitis	0.86
Caregivers’ oral health attitudes	Caregivers’ oral health attitudes	1.00
Caregivers’ oral health practices	Caregivers’ frequencies of sugary snack intake	0.77
	Caregiver’s frequency of toothbrushing	0.48
Children’s oral health practices	Children’s frequencies of sugary snack intake	0.77
	Children’s frequencies of toothbrushing	0.35
	Age of beginning to brush teeth	0.35
Children’s caries	dmft	1.00

## Discussion

The results showed that this study’s theoretical model adequately fitted the data. The findings indicated that the factors influencing dental caries in 5-year-old Chinese children in Guangzhou and the interrelationship between these factors could be explained by the model. Specifically, socioeconomic factors influenced children’s oral health practices through the impact of their caregivers’ oral health knowledge and practices; caregivers’ oral health knowledge affected children’s oral health practices mediated by the caregivers’ oral health attitudes and practices; and finally, children’s oral health practices were directly linked to their dental caries. However, our findings did not guarantee the universal application of this model. The model is hypothetical with regard to the correlations between children’s dental caries status and their determinants, which are supported by the literature and the results from the present study.

The KABP model is composed of knowledge (K), attitude (A), belief (B) and practice (P), but in reality, belief is very similar to attitude; therefore, the KABP model is more specifically a KAP model [14]. The KAP model is always applied in oral health promotion through oral health education [15, 16]. Caregivers have been reported to influence children’s oral health practices by the KAP pathway [7]. In this study, it was found that caregivers’ oral health knowledge affected children’s oral health practices through the caregivers’ oral health attitudes. At the same time, caregivers’ oral health knowledge also influenced children’s oral health practices through the caregivers’ own oral health practices. These results showed that caregivers’ oral health knowledge could impact children’s oral health practices by two pathways. The caregivers’ own dental hygiene habits are important for the children’s oral health practices, and they can be role models for children’s practices in daily life [17, 18]. The coefficient for the path between children’s oral health practices and children’s dental caries was also highly significant, and children with poor oral health practices had more teeth with caries, which revealed that children’s poor oral health practices can be a risk factor for dental caries [19, 20]. These findings have important applications for policy making regarding children’s oral health, and caregivers should be made to realize that they are role models for their children; that is, more attention should be focused on caregivers, including their oral health knowledge, attitudes and practices.

Social structure affects health via interlinking material and psychosocial and behavioral pathways [6], and socioeconomic factors might influence oral health by oral health behaviors or preventive interventions [21]. The present study demonstrated that socioeconomic factors influenced children’s dental caries through their caregivers’ oral health knowledge, attitudes and behaviors and also influenced children’s oral health behaviors, findings that were similar to those reported by Gao *et al.* [7].

Although children’s dental service utilization was removed in the final model, an additional explanation is discussed. In this study, SES was thought to directly or indirectly impact on dental service utilization via oral health knowledge and attitudes [7], but these results were not found with the primary model. It is probable that in our study children’s dental service utilization was dissimilar to that in previous studies. In our study, 30.7% of the children had utilized dental care services, but 85.1% of them visited the dentist solely for fixing dental problems [8]. This result indicates that the relationship between knowledge, attitudes and behaviors is not as simple as that described in the KAB model. These relationships could deviate from each other, and consequently, people with poorer dental knowledge could hold very positive attitudes toward oral health whereas people with positive oral health attitudes could have poor dental behaviors [9]. Furthermore, in this study children’s dental visit was positively related to their caries status, indicating that caries-related problems were probably the reasons for the children’s dental visits.

Methodological strengths and limitations should be considered for the findings in this study. Strengths: First, the SEM method was applied to analyze the direct and indirect effects of oral health risk factors and to reveal the relationships between these risk factors. SEM is superior to multiple regression modeling which can only show the direct effects of oral health risk factors would be shown. Second, a large random sample that contained a variety of socioeconomic groups was used in this study, and the sample was representative. Limitations: First, caries is a disease caused by multiple factors [13], and studies on the roles of genetics, biology, social environment, physical environment, health-influencing behaviors, and medical care are critical for a complete understanding of their influences on oral health; the present study merely explained some of these influences. Second, because the development of caries is chronic and progressive and because the cross-sectional data in this study precluded making inferences about the causal and successive directions between risk factors and children’s dental caries, future work on this topic should consider using a longitudinal approach.

## Conclusion

In conclusion, findings from this study have important applications for policy making with regard to promoting children’s oral health, and caregivers should be made to realize that they are role models for their children; in that area, more attention should be focused on caregivers, including their oral health knowledge, attitudes and practices. Additionally, public education programs should be advocated more broadly, and oral health care service systems should be improved in areas that are socioeconomically disadvantaged.

## Abbreviations

AGFI: Adjusted goodness of fit index
CFI: Comparative fit index
GFI: Goodness of fit index
KAP: Knowledge, attitude and practice
NFI: Normed fit index
RMSEA: Root mean square error of approximation
SEM: Structural equation modeling
SES: Socioeconomic status.
